# Changes in Disease Severity and Outcomes Among Electively Admitted Cirrhosis Patients During the COVID-19 Era

**DOI:** 10.3390/healthcare14091255

**Published:** 2026-05-06

**Authors:** Melania Veronica Ardelean, Dana Roxana Buzas, Alin Viorel Istodor, Paul Ciubotaru, Vlad Ivan, Norina Simona Basa, Daniel Florin Lighezan, Dan Iliescu, Ovidiu Florin Ardelean

**Affiliations:** 1 Department V, Internal Medicine I Discipline of Medical Semiology I, “Victor Babes” University of Medicine and Pharmacy, Eftimie Murgu Sq. No 2, 300041 Timisoara, Romania; ardelean.melania@umft.ro (M.V.A.); ivan.vlad@umft.ro (V.I.); basa.simona@umft.ro (N.S.B.); dlighezan@umft.ro (D.F.L.); 2 Center of Advanced Research in Cardiology and Hemostasology, “Victor Babes” University of Medicine and Pharmacy, Eftimie Murgu Sq. No 2, 300041 Timisoara, Romania; 3 First Department of Surgery, First Discipline of Surgical Semiology and Thoracic Surgery, “Victor Babes” University of Medicine and Pharmacy, Eftimie Murgu Sq. No 2, 300041 Timisoara, Romania; dan.iliescu@umft.ro (D.I.); ardelean.ovidiu@umft.ro (O.F.A.); 4 Department of General Surgery, Emergency City Hospital, 300254 Timisoara, Romania

**Keywords:** liver cirrhosis, elective hospitalization, COVID-19 pandemic, disease severity, portal hypertension, healthcare disruption, delayed decompensation, clinical outcomes

## Abstract

**Background**: Coronavirus disease 2019 (COVID-19), caused by the SARS-CoV-2 virus, had profound primary effects on global health and secondary effects through widespread disruption of healthcare systems, limiting access to elective medical services essential for the management of chronic diseases such as liver cirrhosis. Elective hospitalizations play a key role in disease monitoring, prevention of complications, and therapeutic optimization. This study aimed to evaluate the impact of the pandemic on the clinical profile, disease severity, and outcomes of patients electively admitted with liver cirrhosis across three periods: pre-pandemic, pandemic, and post-pandemic. **Methods:** This retrospective, single-center cohort study included 248 adult patients electively admitted with a primary diagnosis of liver cirrhosis between February 2018 and February 2024. Patients were stratified according to admission period. Data on demographics, clinical presentation, etiology, decompensation markers, severity scores (Child–Pugh, Baveno), procedures, and hospitalization outcomes were analyzed. **Results**: A total of 248 patients were included, with a significant reduction in elective admissions during the pandemic (23.0% vs. 46.4% pre-pandemic), followed by partial recovery post-pandemic (30.6%) (*p* = 0.031). A higher proportion of urban patients was observed during the pandemic (70.2%, *p* = 0.004). Disease severity increased during the pandemic, with a higher prevalence of Child–Pugh C (17.5%) and Baveno stage 6 (10.5%), whereas post-pandemic data showed improvement (Child–Pugh C: 6.57%; no Baveno stage 6; *p* = 0.004). Ascites (47.4%) and paracentesis (21.1%) peaked post-pandemic (*p* = 0.012; *p* = 0.003). Endoscopic activity decreased during the pandemic (22.8%, *p* = 0.017), while interventional procedures were more frequent (8.8%, *p* = 0.045). Transfusion requirements (17.5%, *p* = 0.001) and hospitalization costs (€467.08, *p* = 0.01) were highest during the pandemic, while no deaths were recorded post-pandemic. In-hospital mortality was observed in 1.7% of patients during the pre-pandemic period and increased to 3.5% during the pandemic period, while no deaths were recorded post-pandemic. **Conclusions:** The COVID-19 pandemic significantly altered elective cirrhosis care, leading to reduced admissions, increased disease severity, and higher resource utilization. Although partial recovery was observed post-pandemic, persistent evidence of delayed decompensation underscores the importance of maintaining continuity in elective hepatology services.

## 1. Introduction

The coronavirus disease 2019 (COVID-19), caused by the novel severe acute respiratory syndrome coronavirus 2 (SARS-CoV-2), rapidly developed into a global health crisis that placed an extraordinary burden on healthcare systems worldwide, pushing many to the brink of collapse [1,2,3]. Beyond its direct clinical impact, the pandemic also led to widespread disruptions in healthcare delivery, affecting nearly all medical specialties and disease areas, particularly those requiring continuous monitoring and timely interventions [4,5]. In this context, the management of chronic liver diseases such as cirrhosis—conditions associated with significant morbidity and mortality—was especially vulnerable to these systemic disruptions.

Liver cirrhosis remains a major contributor to global mortality, accounting for a substantial proportion of deaths related to chronic liver disease. Recent estimates indicate that over one million deaths annually are attributable to cirrhosis and its complications, most commonly driven by portal hypertension and hepatocellular carcinoma [6,7]. While the overall prevalence in Europe is estimated at approximately 0.3% of the adult population, the burden is disproportionately higher in Eastern European countries, where alcohol consumption and chronic viral hepatitis play a dominant role [8]. In Romania, this pattern is particularly evident, with chronic liver disease representing a persistent public health challenge and mortality rates among the highest in the European Union [9].

Cirrhosis arises from multiple etiological factors, including chronic hepatitis B and C infection, alcohol-related liver disease, and non-alcoholic fatty liver disease. In clinical practice across Eastern Europe, alcohol-related etiology continues to predominate among hospitalized patients [8,10]. The disease is defined by progressive fibrosis and irreversible distortion of hepatic structure, leading to portal hypertension and impaired liver function. As cirrhosis evolves, complications such as ascites, variceal bleeding, hepatic encephalopathy, and hypersplenism become increasingly frequent, contributing to both clinical deterioration and increased demand on healthcare resources [11,12].

The effective management of cirrhosis depends not only on acute interventions but also on consistent access to planned medical care. Elective hospitalizations provide an essential framework for disease surveillance, therapeutic adjustment, and early management of complications [13]. However, the emergence of the COVID-19 pandemic led to a rapid restructuring of healthcare priorities, with substantial resources being redirected toward the treatment of patients with SARS-CoV-2 infection [4,14,15]. This shift resulted in reduced availability of routine medical services, including elective admissions and non-urgent procedures, as healthcare systems sought to balance infection control with capacity constraints [16,17]. As a result, continuity of care for chronic liver disease patients was significantly disrupted [18,19].

Accumulating evidence suggests that these changes translated into measurable clinical consequences. Several reports have described a decline in hospital admissions and endoscopic activity among patients with chronic liver disease during the pandemic period [20,21]. At the same time, reduced healthcare accessibility and delays in seeking medical attention were associated with more advanced disease at presentation and an increased burden of complications [22]. In addition, concerns regarding exposure to SARS-CoV-2 contributed to postponement of medical evaluation, further exacerbating delays in diagnosis and treatment [23,24].

Given these circumstances, evaluating the impact of the COVID-19 pandemic on the clinical profile and management of patients with liver cirrhosis requiring elective hospitalization is of particular importance. The present study aims to comparatively analyze demographic characteristics, clinical presentation, complications, disease severity, and hospitalization-related outcomes among patients electively admitted with liver cirrhosis across three distinct periods: pre-pandemic, pandemic, and post-pandemic. By identifying changes in patient characteristics and patterns of healthcare utilization, this study seeks to provide insight into how the pandemic influenced access to elective hepatology care and the clinical profile of patients requiring planned hospitalization. Furthermore, the findings are intended to provide essential evidence that may serve as a foundation for addressing potential challenges faced by healthcare systems during future pandemics or similar large-scale disruptions.

## 2. Materials and Methods

### 2.1. Study Design and Population

This retrospective, observational cohort study was conducted at the Department of Gastroenterology of the Municipal Clinical Emergency Hospital in Timișoara, Romania. The analysis focused on adult patients electively hospitalized with liver cirrhosis as the primary diagnosis. All relevant clinical, biochemical, and demographic data were obtained from electronic medical records and analyzed systematically.

The study spanned a six-year period, from 26 February 2018 to 25 February 2024. To explore the impact of the COVID-19 pandemic on cirrhosis management, the study interval was divided into three phases corresponding to major epidemiological milestones in Romania:

- pre-pandemic phase (26 February 2018–25 February 2020),
- pandemic phase (26 February 2020–25 February 2022),
- post-pandemic phase (26 February 2022–25 February 2024).

These intervals were defined based on the date of the first confirmed SARS-CoV-2 case in Romania (26 February 2020) and the subsequent lifting of most national restrictions (8 March 2022).

### 2.2. Eligibility Criteria

Patients were eligible for inclusion if they met the following criteria:

- adult age (≥18 years)
- confirmed diagnosis of liver cirrhosis, regardless of etiology, established based on clinical, laboratory, imaging, and/or endoscopic findings;
- elective hospital admission during the predefined study periods;
- liver cirrhosis as the primary reason for hospitalization, rather than other concomitant conditions
- absence of confirmed or active SARS-CoV-2 infection at the time of admission;
- absence of typical COVID-19 symptoms within the seven days preceding hospitalization.

Patients with confirmed COVID-19 infection or with clinical suspicion of SARS-CoV-2 infection were excluded from the study. Due to the well-established adverse prognostic impact of COVID-19 in patients with cirrhosis, these individuals were managed in designated isolation units according to separate institutional protocols and were therefore not included in the present analysis.

After applying these criteria, a final cohort of 248 patients was retained for statistical analysis.

### 2.3. Data Collection and Study Variables

Several categories of variables were analyzed.

Demographic characteristics included age, sex, and area of residence (urban or rural).

Clinical presentation was assessed based on the symptoms reported at admission, including abdominal pain, upper gastrointestinal bleeding (UGIB; melena or hematemesis), lower gastrointestinal bleeding (LGIB; rectorrhagia or hematochezia), jaundice (reported by patients as yellowing of the skin or sclera), lower limb edema (LLE), or other complaints with a frequency below 5%.

Etiology of cirrhosis was categorized as alcoholic, viral, mixed, or other causes.

Disease severity was evaluated using established prognostic indices:

- Child–Pugh score
- Baveno staging system

Procedural indicators comprised the need for therapeutic endoscopic interventions, such as variceal band ligation or endoscopic hemostasis.

Markers of hepatic decompensation included the presence of ascites, splenomegaly, esophageal varices, hepatic encephalopathy, thrombocytopenia, and the requirement for therapeutic paracentesis.

Supportive management variables included the requirement for blood transfusions and the duration of hospitalization.

Economic parameters were assessed by calculating the total cost of hospitalization per patient.

### 2.4. Ethical Considerations

The study protocol was approved by the Ethics Committee of the Municipal Clinical Emergency Hospital in Timișoara (Approval No. E-245/21 January 2026). All procedures were conducted in accordance with the principles of the Declaration of Helsinki and with national regulations governing biomedical research involving human subjects.

### 2.5. Statistical Analysis

All statistical analyses were performed using IBM SPSS Statistics version 25.0 (IBM Corp., Armonk, NY, USA). A *p* value < 0.05 was considered statistically significant.

Continuous variables were expressed as mean ± standard deviation (SD), while categorical variables were presented as absolute numbers and percentages.

The distribution of continuous variables was evaluated using the Shapiro–Wilk test. Age showed normal distribution and was analyzed using parametric methods (Student’s *t*-test or one-way ANOVA, as appropriate), with Levene’s test for homogeneity of variances and Bonferroni correction for multiple comparisons when applicable. In contrast, length of hospital stay and hospitalization costs showed non-normal distribution and were analyzed using non-parametric tests (Mann–Whitney U test or Kruskal–Wallis test, as appropriate). All tests were two-tailed.

Categorical variables were analyzed using the Chi-square test, with Fisher’s exact test applied when expected cell counts were below 5.

Missing data were handled through listwise deletion, without imputation.

## 3. Results

Between 26 February 2018 and 25 February 2024, a total of 248 patients with a primary diagnosis of liver cirrhosis were electively admitted to the Department of Gastroenterology of the Municipal Hospital of Timișoara. A statistically significant variation in the number of hospitalizations was observed across the three evaluated periods (*p* = 0.031). Of the total cohort, 115 patients (46.4%) were admitted during the pre-pandemic period, 57 (23.0%) during the pandemic, and 76 (30.6%) in the post-pandemic interval.

Significant differences were observed across the three periods with regard to patients’ area of residence. In contrast, no statistically significant differences were identified in terms of mean age or the proportion of male patients between the evaluated intervals. The detailed distribution of these variables is presented in Table 1.

### 3.1. Symptoms at Presentation

Abdominal pain was the most frequently reported symptom at admission, being documented in 130 patients (52.4%). The distribution across the study periods included 67 cases (58.3%) in the pre-pandemic interval, 27 cases (47.4%) during the pandemic, and 36 cases (47.4%) in the post-pandemic period. These differences were not statistically significant (*p* = 0.231).

The distribution of the main clinical reasons for presentation across the three study periods is summarized in Table 2.

An additional finding was the higher prevalence of jaundice among patients from rural areas compared with those from urban settings (66.7% vs. 33.3%, *p* = 0.004).

### 3.2. Clinical Manifestations

Hepatic encephalopathy remained relatively stable across the three study periods, being identified in 28 patients (24.3%) in the pre-pandemic phase, 16 patients (28.1%) during the pandemic, and 18 patients (23.7%) in the post-pandemic interval, without statistically significant differences (*p* = 0.826).

In contrast, significant differences were observed in the prevalence of thrombocytopenia across the evaluated periods (*p* = 0.005). Low platelet counts were documented in 75 patients (65.2%) in the pre-pandemic group, 35 patients (61.4%) during the pandemic, and 64 patients (84.2%) in the post-pandemic period.

The etiology of cirrhosis did show statistically significant variation across the three time intervals. Detailed data are provided in Table 3.

The prevalence of splenomegaly differed significantly across the three study periods (*p* = 0.009). Splenic enlargement was documented in 48 patients (41.7%) in the pre-pandemic period, 27 patients (47.4%) during the pandemic, and 18 patients (23.7%) in the post-pandemic interval.

The prevalence of esophageal varices did not differ significantly across the three study periods (*p* = 0.311). Esophageal varices were identified in 42 patients (36.5%) during the pre-pandemic period, 17 patients (29.8%) during the pandemic, and 20 patients (26.3%) in the post-pandemic interval. Only a difference between the pre-pandemic and post-pandemic period was reported (*p* = 0.024). Nevertheless, a significantly higher proportion of patients with esophageal varices originated from rural areas compared with urban areas (57% vs. 43%, *p* = 0.02).

The prevalence of ascites was highest in the post-pandemic period, affecting 36 patients (47.4%), compared with 35 patients (30.4%) in the pre-pandemic phase and 14 patients (24.6%) during the pandemic. These differences were statistically significant (*p* = 0.012).

Similarly, the proportion of patients requiring paracentesis differed significantly across the three periods, being reported in 10 patients (8.7%) in the pre-pandemic group, 2 patients (3.5%) during the pandemic, and 16 patients (21.1%) in the post-pandemic interval (*p* = 0.003).

Significant differences were observed in the proportion of patients undergoing gastroscopy during hospitalization across the three study periods (*p* = 0.017). The lowest rate was recorded during the pandemic (22.8%), compared with 45.2% in the pre-pandemic interval and 36.8% in the post-pandemic period.

Moreover, the need for interventional endoscopic procedures varied significantly between periods (*p* = 0.045). A total of 6 cases (5.2%) were reported in the pre-pandemic period and 5 cases (8.8%) during the pandemic, while no such procedures were performed in the post-pandemic interval. Notably, all 11 patients requiring interventional endoscopy originated from rural areas.

Cirrhosis severity, assessed using the Child–Pugh classification, showed significant differences across the three periods, with a higher proportion of patients categorized as severe during the pandemic. Detailed data are summarized in Table 4.

The Baveno score, used to assess the severity of cirrhosis, also showed significant variation across the evaluated time periods. The detailed distribution of these findings is presented in Table 5.

### 3.3. Hospitalization

Length of hospital stay was also evaluated as a key parameter. No statistically significant differences were observed across the three study periods (Kruskal–Wallis, *p* = 0.471). The mean duration of hospitalization was 4.84 ± 1.87 days in the pre-pandemic period, 4.51 ± 1.71 days during the pandemic, and 4.76 ± 1.35 days in the post-pandemic interval.

The requirement for blood transfusions during hospitalization also varied across the study periods. Transfusions were administered to 8 patients (7%) in the pre-pandemic period and to 10 patients (17.5%) during the pandemic, whereas no patients required transfusion in the post-pandemic interval. A statistically significant difference was observed between the pandemic and post-pandemic periods (*p* = 0.001).

Average hospitalization costs were €417.63 ± 182.35 in the pre-pandemic period, increasing to €467.08 ± 236.27 during the pandemic and subsequently decreasing to €357.47 ± 120.78 in the post-pandemic interval. Overall, hospitalization costs were significantly higher during the pandemic compared with the other two periods (Kruskal–Wallis, *p* = 0.01), representing an increase of 11.6% relative to the pre-pandemic phase and a decrease of 23.55% in the post-pandemic period.

A total of four deaths were recorded during the study period, with two occurring during the pandemic and two during the pre-pandemic interval. No deaths were reported in the post-pandemic period.

## 4. Discussion

### 4.1. Impact of the COVID-19 Pandemic on Elective Cirrhosis Admissions

The present study demonstrates a clear impact of the COVID-19 pandemic on elective hospital admissions for patients with liver cirrhosis. A substantial decline in admissions was observed during the pandemic, from 115 cases (46.4%) in the pre-pandemic period to 57 cases (23.0%), followed by a partial recovery to 76 cases (30.6%) thereafter. These findings are in line with previously reported data showing a widespread reduction in non-urgent hospital activity during COVID-19, largely driven by the reallocation of healthcare resources and the implementation of infection control measures that limited access to planned care [25,26,27]. In the context of cirrhosis, where elective admissions are essential for surveillance, therapeutic optimization, and prevention of decompensation, such disruptions may have downstream consequences on disease evolution and clinical presentation [11,13].

Notably, the demographic profile of the study cohort remained stable throughout the three periods, with no significant variations in age or sex distribution. This consistency suggests that the differences observed across time are unlikely to reflect true epidemiological changes in cirrhosis, but rather shifts in healthcare access and utilization. In contrast, a clear redistribution was observed in terms of patients’ area of residence, with a marked predominance of individuals from urban settings during the pandemic (70.2% compared to 43.5% in the pre-pandemic period and 55.3% post-pandemic). This trend most likely reflects the reduced accessibility of tertiary healthcare services for rural populations during periods characterized by mobility restrictions and increased strain on healthcare systems.

This interpretation is further supported by the more severe clinical profile observed among patients originating from rural areas. Advanced manifestations of liver disease, including jaundice and esophageal varices, were significantly more frequent in this group (66.7% vs. 33.3%, *p* = 0.004 and 57% vs. 43%, *p* = 0.02, respectively). In addition, all patients who required interventional endoscopic procedures were from rural environments. Together, these findings indicate a pattern of delayed presentation and more advanced portal hypertension among patients with limited access to specialized care. Similar disparities have been reported in previous studies, highlighting the disproportionate impact of the pandemic on vulnerable populations and its role in delaying the diagnosis and management of chronic liver disease [28,29,30].

### 4.2. Clinical Presentation and Evolution of Complications

Abdominal pain was the most frequently reported symptom at presentation, affecting 52.4% of patients, without significant variation across the three study periods (58.3% vs. 47.4% vs. 47.4%, *p* = 0.231). This relative stability suggests that abdominal pain is not a reliable indicator of disease severity in cirrhosis, but rather reflects its nonspecific nature. In cirrhotic patients, abdominal pain may result from multiple underlying mechanisms, including ascites-related distension, portal hypertension, or coexisting gastrointestinal conditions [28,31]. Consequently, its consistent prevalence across periods indicates a limited sensitivity to changes in healthcare access or system-level disruptions, unlike more specific clinical manifestations.

In contrast, several indicators more directly related to disease progression showed notable temporal variation. The increased frequency of lower gastrointestinal bleeding during the pandemic (7% vs. 1.7% pre-pandemic), followed by its absence in the post-pandemic period (*p* = 0.027), may reflect delayed presentation and restricted access to medical care—patterns widely documented during the COVID-19 period [23,32,33]. At the same time, the progressive rise in lower limb edema, reaching 18.4% in the post-pandemic phase (*p* = 0.014), suggests a growing burden of chronic decompensation. This trend is likely attributable to interruptions in routine follow-up and delays in therapeutic optimization during the pandemic [30].

Markers of hepatic dysfunction, including jaundice (17.4% vs. 14% vs. 18.4%, *p* = 0.788) and hepatic encephalopathy (24.3% vs. 28.1% vs. 23.7%, *p* = 0.826), remained relatively stable. This may reflect the fact that severe decompensations, particularly encephalopathy, are more frequently managed in emergency settings rather than through elective admissions [11,28].

A more nuanced pattern was observed for indicators of portal hypertension. The significant increase in thrombocytopenia in the post-pandemic period (84.2% vs. 65.2% and 61.4%, *p* = 0.005), in parallel with a decrease in splenomegaly (23.7% vs. 41.7% and 47.4%, *p* = 0.009), highlights the complex pathophysiology of thrombocytopenia in cirrhosis. Beyond hypersplenism, mechanisms such as reduced thrombopoietin production and bone marrow suppression play a significant role, which may persist despite clinical improvement [34,35].

The marked differences in cirrhosis etiology observed across the pre-pandemic, pandemic, and post-pandemic periods in our cohort reflect patterns that have been increasingly reported worldwide during the COVID-19 era. In our study, alcohol-associated cirrhosis showed a substantial decline from 72.2% in the pre-pandemic period to 50.9% during the pandemic and 47.4% post-pandemic, while other etiologies increased markedly (from 3.5% to over 25%, *p* < 0.001). Similar trends have been described in international cohorts, although they appear paradoxical in the context of reported increases in alcohol consumption during lockdown periods [36,37]. This apparent discrepancy has been attributed to altered healthcare-seeking behavior, with patients with alcohol-related liver disease being less likely to engage in elective care and more likely to present with acute decompensation requiring emergency admission [27].

In parallel, studies have highlighted a relative increase in non-alcohol-related etiologies, including viral and metabolic liver disease, among patients accessing structured care during the pandemic [38,39]. This shift has been linked to the prioritization of patients already integrated into follow-up programs and to the reorganization of healthcare systems, which limited access to elective services for more vulnerable or socially disadvantaged populations [22]. Furthermore, widespread disruption of hepatology services, including reduced outpatient visits and endoscopic procedures, has contributed to delayed diagnosis and changes in the clinical profile of hospitalized patients [28,30].

Taken together, these findings support the interpretation that the etiological redistribution observed in our cohort does not reflect a true change in the epidemiology of cirrhosis, but rather a pandemic-driven shift in healthcare access, patient selection, and patterns of hospital admission, with important implications for the interpretation of clinical outcomes during this period.

### 4.3. Portal Hypertension, Endoscopic Activity, and Disease Severity

Findings related to portal hypertension provide important insight into how both disease monitoring and patient selection were affected during the pandemic. Although the overall prevalence of esophageal varices remained relatively stable across the three periods (36.5% vs. 29.8% vs. 26.3%, *p* = 0.311), the observed decrease from the pre- to the post-pandemic phase (*p* = 0.024) is more likely attributable to reduced detection rather than a true reduction in disease burden. This interpretation is consistent with the well-documented limitations in endoscopic access during the COVID-19 period [28,29].

This hypothesis is further supported by the substantial decline in gastroscopy rates during the pandemic (22.8% compared to 45.2% pre-pandemic and 36.8% post-pandemic, *p* = 0.017). Similar trends have been described in the literature, where endoscopic procedures were largely restricted to urgent indications in order to minimize infection risk and reallocate healthcare resources [21,28]. As a consequence, complications related to portal hypertension may have been underdiagnosed during this interval.

Interestingly, despite the overall reduction in endoscopic activity, the proportion of interventional procedures was highest during the pandemic (8.8% vs. 5.2% vs. 0%, *p* = 0.045). This pattern suggests a shift toward severity-driven care, in which only patients with high-risk features or active bleeding were prioritized for endoscopic intervention. Such findings are in line with previous reports indicating that, during the pandemic, endoscopic services were predominantly reserved for urgent or life-threatening conditions [21,39]. Ascites and paracentesis followed a distinct trajectory, both peaking in the post-pandemic period (47.4% and 21.1%, respectively), significantly higher than in earlier intervals (*p* = 0.012 and *p* = 0.003). This likely reflects delayed presentation and a “catch-up” effect, with patients returning to care in a more decompensated state after restricted access, as previously reported in chronic liver disease cohorts [28,30].

Severity scores further support this pattern. The pandemic period showed a higher proportion of Child–Pugh C patients (17.5%), whereas the post-pandemic period demonstrated improvement (6.57%). More notably, Baveno stage 6 was present only during the pandemic (10.5%) and absent thereafter, while Baveno stage 3 increased to 26.3% post-pandemic (*p* = 0.004). This shift toward lower severity stages suggests partial restoration of earlier diagnosis and follow-up once healthcare access improved [11].

### 4.4. Hospital Outcomes and Evidence of Post-Pandemic Recovery

Hospital outcomes further reflect the increased burden of disease during the pandemic. Although length of stay remained stable (*p* = 0.471), transfusion requirements were significantly higher during the pandemic (17.5% vs. 7% pre-pandemic vs. 0% post-pandemic, *p* = 0.001), indicating more severe or unstable clinical presentations at admission.

A similar trend was observed for hospitalization costs, which peaked during the pandemic (€467.08 vs. €417.63 pre-pandemic and €357.47 post-pandemic, *p* = 0.01). This increase likely reflects both greater clinical complexity and system-level inefficiencies, including resource reallocation and infection control measures, as described in multiple healthcare analyses during COVID-19 [27,28].

The post-pandemic period was associated with more favorable clinical outcomes, notably the absence of transfusion requirements and the lack of recorded in-hospital mortality, compared with two deaths observed in each of the preceding periods. Although these findings should be interpreted with caution, they are in line with the observed improvement in disease severity, particularly as reflected by Baveno staging.

Taken together, these results suggest a distinct three-phase pattern. The pre-pandemic period was characterized by relative stability, followed by a pandemic phase marked by reduced healthcare access and increased disease severity among admitted patients. This was succeeded by a partial recovery phase, in which overall outcomes improved, although signs of delayed decompensation remained evident. These observations highlight the critical importance of preserving access to elective hepatology services, as even short-term disruptions may have sustained consequences on disease progression and healthcare utilization [11,28].

### 4.5. Study Limitations

This study has several limitations that should be acknowledged. Its retrospective, single-center design may limit the generalizability of the findings, as patterns of elective admission and access to hepatology care may vary across institutions. Furthermore, by focusing exclusively on electively admitted patients, the study does not capture individuals presenting emergently, potentially underestimating the full burden of cirrhosis during the pandemic.

Changes in healthcare-seeking behavior, admission policies, and access to diagnostic procedures during COVID-19 represent additional sources of bias that could have influenced both patient selection and clinical presentation. In particular, reduced availability of endoscopic evaluation may have led to underdiagnosis of certain complications. Finally, the economic analysis was limited to direct hospitalization costs and did not include indirect or societal expenses.

Despite these limitations, the study provides valuable insight into how disruptions in elective hepatology care influenced disease presentation and outcomes across the pandemic timeline.

## 5. Conclusions

This study demonstrates that the COVID-19 pandemic significantly impacted the management of patients with liver cirrhosis requiring elective hospitalization, leading to reduced admissions, changes in clinical presentation, and shifts in disease severity. While demographic characteristics remained stable, the pandemic period was marked by limited access to diagnostic procedures, a higher proportion of severe cases, and increased healthcare resource utilization. Notably, disparities between urban and rural populations became more pronounced, with rural patients presenting with more advanced disease. A significant shift in cirrhosis etiology was observed during the pandemic, with a reduction in alcohol-associated cirrhosis and a relative increase in other etiologies.

The post-pandemic period showed partial recovery, reflected by increased admission rates, improved severity profiles, and better clinical outcomes. However, the persistence of complications such as ascites and increased need for paracentesis suggests a delayed burden of disease following disrupted care. These findings highlight the importance of maintaining continuity in elective hepatology services, as even temporary disruptions may have lasting effects on disease progression and patient outcomes.

## Figures and Tables

**Table 1:** Demographic aspects.

Variables	Pre-Pandemic (*n* = 115)	Pandemic (*n* = 57)	Post-Pandemic (*n* = 76)	*p*
Age (years, M ± SD)	59.93 ± 10.27	59.66 ± 10.39	58.72 ± 10.65	0.073
Gender Male	109 (79%)	79 (82.3%)	89 (73.6%)	0.586
Environment				
Urban	50 (43.5%)	40 (70.2%)	42 (55.3%)	0.004
Rural	65 (56.5%)	17 (29.8%)	34 (44.7%)	

M = mean; SD = Standard Deviation.

**Table 2:** Clinical reasons for presentation.

Variables	Pre-Pandemic (*n* = 138)	Pandemic (*n* = 96)	Post-Pandemic (*n* = 121)	*p*
UGIB	4 (3.5%)	3 (5.3%)	0	0.163
LGIB	2 (1.7%)	4 (7%)	0	0.027
LLE	6 (5.2%)	6 (10.5%)	14 (18.4%)	0.014
Jaundice	20 (17.4%)	8 (14%)	14 (18.4%)	0.788

Comparisons between groups were performed using the Chi-square test or Fisher’s exact test, as appropriate. Fisher’s exact test was applied when the expected cell counts were <5.

**Table 3:** Cirrhosis etiology.

Cirrhosis Etiology	Pre-Pandemic (*n* = 115)	Pandemic (*n* = 57)	Post-Pandemic (*n* = 76)	*p*
Alcoholic	83 (72.2%)	29 (50.9%)	36 (47.4%)	<0.001
Mixed	12 (10.4%)	2 (3.5%)	6 (7.9%)	
Viral	16 (13.9%)	10 (17.5%)	14 (18.4%)	
others	4 (3.5%)	16 (28.1%)	20 (26.3%)	

Comparisons between groups were performed using the Chi-square test or Fisher’s exact test, as appropriate. Fisher’s exact test was applied when the expected cell counts were <5.

**Table 4:** Child Pugh variation across the 3 periods.

Child Pugh Class	Pre-Pandemic (*n* = 115)	Pandemic (*n* = 57)	Post-Pandemic (*n* = 76)	*p*
A	37 (32.2%)	26 (45.6%)	30 (39.47%)	0.021
B	62 (53.9%)	21 (36.8%)	41 (53.94%)	
C	16 (13.9%)	10 (17.5%)	5 (6.57%)	

Comparisons between groups were performed using the Chi-square test or Fisher’s exact test, as appropriate. Fisher’s exact test was applied when the expected cell counts were <5.

**Table 5:** Baveno variation across the 3 periods.

BAVENO	Pre-Pandemic (*n* = 115)	Pandemic (*n* = 57)	Post-Pandemic (*n* = 76)	*p*
3	10 (8.7%)	10 (17.5%)	20 (26.3%)	0.004
4	81 (70.4%)	34 (59.6%)	42 (55.3%)	
5	18 (15.7%)	7 (12.3%)	14 (18.4%)	
6	6 (5.2%)	6 (10.5%)	0	

Comparisons between groups were performed using the Chi-square test or Fisher’s exact test, as appropriate. Fisher’s exact test was applied when the expected cell counts were <5.

## Data Availability

Data available on request due to patient confidentiality, privacy protection regulations (including GDPR), and institutional policies governing access to clinical data. Further inquires can be made upon reasonable request from the corresponding authors.
